# Maternal nativity and preterm birth disparities in twin pregnancies: A nationwide population‐based study

**DOI:** 10.1002/pmf2.70361

**Published:** 2026-07-20

**Authors:** Hiba J. Mustafa, Jana Karam, William A. Grobman, Kevin L. Moss, Jasmine Johnson, Maged M. Costantine, Heather A. Frey, Moti Gulersen, Vincenzo Berghella, Alireza A. Shamshirsaz

**Affiliations:** ^1^ The Fetal Center at Riley Children's and Indiana University Health Division of Maternal‐Fetal Medicine Indiana University School of Medicine Indianapolis Indiana USA; ^2^ Obstetrics and Gynecology Lebanese American University School of Medicine Beirut Lebanon; ^3^ Department of Obstetrics and Gynecology Brown University Medical School Providence Rhode Island USA; ^4^ Department of Biostatistics and Health Data Science Indiana University School of Medicine Indianapolis Indiana USA; ^5^ Division of Maternal‐Fetal Medicine Department of Obstetrics and Gynecology The Ohio State University Columbus Ohio USA; ^6^ Division of Maternal‐Fetal Medicine Thomas Jefferson University Philadelphia Pennsylvania USA; ^7^ Comprehensive Fetal Care Center at Dell Children's Dell Children's Medical Center Austin Texas USA

**Keywords:** immigrants, pregnancy outcome, preterm birth, twins

## Abstract

**Objective:**

To evaluate whether maternal nativity (US born vs. non–US born) is associated with preterm birth (PTB) risk in individuals with twin pregnancies and to assess whether this association varies across racial and ethnic groups.

**Methods:**

This cross‐sectional study used the National Center for Health Statistics and Centers for Disease Control and Prevention (CDC) natality dataset (2016–2023). This analysis included individuals aged 15–44 years with live‐born twins residing in the United States. Maternal nativity (US born vs. non–US born) was stratified by race and ethnicity. Covariates included maternal age, education, insurance, prenatal care, smoking, prepregnancy body mass index, diabetes, hypertension, and year of birth. PTB (<37 weeks) was further categorized as extremely (<29 weeks), moderately (29–33weeks), and late (34–36 weeks). Binary and multinomial logistic regressions were used to calculate adjusted odds ratios (aORs) and 95% confidence intervals (CIs).

**Results:**

Among 467,446 twin pregnancies, 282,754 (60.5%) were preterm. Of these, 191,972 (41.1%) were classified as late preterm (34 to <37 weeks), 68,457 (14.6%) as moderately preterm (29 to <34 weeks), and 22,325 (4.8%) as extremely preterm (<29 weeks). Non‐US‐born individuals had lower odds of PTB compared to US‐born counterparts (aOR, 0.88; 95% CI, 0.86–0.89). The protective effect was strongest among non‐US‐born Black individuals (aOR, 0.65; 95% CI, 0.62–0.68). Across PTB subcategories, non‐US‐born individuals had reduced odds of extremely preterm (aOR, 0.85; 95% CI, 0.81–0.88), moderately preterm (aOR, 0.82; 95% CI, 0.80–0.84), and late PTB (aOR, 0.90; 95% CI, 0.88–0.92).

**Conclusions:**

Non‐US‐born individuals had lower odds of PTB compared to US‐born individuals among twin pregnancies, extending prior findings from singleton populations to this high‐risk group. These findings reinforce the role of nativity as an important social determinant of perinatal health. Future work should examine the mechanisms underlying these disparities and the influence of structural and cultural factors on twin birth outcomes.

## 1 INTRODUCTION

Preterm birth (PTB), defined as delivery before 37 weeks of gestation, is a leading cause of neonatal morbidity and mortality worldwide [1]. In 2023, PTB accounted for 10.4% of all live births in the United States, highlighting the ongoing challenge of reducing this risk despite advancements in obstetric care [2]. The consequences of PTB extend beyond the neonatal period, leading to significant long‐term health complications and an increased risk of mortality, particularly for those born extremely preterm [3, 4].

Twin gestation is a well‐established risk factor for PTB, miscarriage, and neonatal morbidity and mortality [5, 6]. Various factors, including short mid‐trimester cervical length, a history of PTB, monochorionicity, prior cervical procedures, and in vitro conception further increase this risk [7], as do maternal complications within pregnancy, such as preeclampsia and gestational diabetes, or fetal factors, such as growth restriction [7].

A growing body of research suggests that maternal nativity (i.e., whether a mother who lives in the United States is born inside or outside the United States) is associated with PTB risk [8, 9, 10]. Studies indicate that, even after adjusting for sociodemographic and medical factors, individuals who live in the United States but were born outside of the United States have a lower risk of PTB than their US‐born counterparts [11, 12]. Nativity may serve as a proxy for various social constructs and structural influences, including citizenship status, acculturation, linguistic proficiency, and exposure to discrimination and xenophobia [13].

The “immigrant paradox” refers to the epidemiologic observation that individuals who are born outside the United States often experience less adverse birth outcomes than their US‐born counterparts despite facing greater overt socioeconomic disadvantages and barriers to healthcare access than US‐born individuals [14, 15]. However, this paradox has not been observed for all conditions, and has been shown to differ by other factors, including country of origin [16, 17].

While the association of maternal nativity has been studied for those with singleton pregnancies, its impact on PTB risk in those with twin pregnancies remains unclear. This study aims to determine whether maternal nativity (US born vs. non–US born) is associated with the risk of PTB in individuals with twin pregnancies and whether these effects vary across different racial and ethnic groups.

## 2 MATERIALS AND METHODS

### 2.1 Study design

We performed a cross‐sectional population study utilizing the National Center for Health Statistics and Centers for Disease Control and Prevention (CDC) natality dataset from January 2016 to December 2023. The natality dataset includes paternal, maternal, prenatal, labor, and obstetric characteristics. The information is derived from the national birth registry and uses the US Standard Certificate for live birth.

### 2.2 Inclusion and exclusion criteria

We included pregnant individuals aged 15–44 years at delivery with live birth twin pregnancies. We excluded those with singletons and higher order multiples; records with missing data on nativity, race/ethnicity, or gestational age (GA) at delivery; outliers of GA at delivery of <20 and >45 weeks; and maternal age <15 and >44 years.

Indiana University Institutional Review Board deemed this study exempt from ethics review and informed consent requirements because it used de‐identified data. We adhered to the Strengthening the Reporting of Observational Studies in Epidemiology (STROBE) guidelines.

### 2.3 Definition of variables and outcomes of interest

The following variables were extracted from the CDC dataset: year of birth, maternal age, nativity (born in/outside of the United States), education (categorized as <high school, high school diploma, any college, bachelor's degree, and advanced degree), insurance (categorized as Medicaid, private, and other), parity, prepregnancy body mass index (BMI), prepregnancy diabetes mellitus (DM), prepregnancy hypertension (HTN), hypertensive disorders of pregnancy (HDP), first‐trimester cigarette smoking, presence of prenatal care, and GA at delivery (categorized as <24, 24–<34, 34–<37, and ≥37 weeks).

Nativity in the CDC data was defined as follows: individuals born in any of the 50 US states were defined as US born. Individuals born outside of the 50 states, which includes US territories, were defined as non–US born by the CDC database [18, 19]. Race‐ethnicity is self‐reported in the CDC Data, and, for this analysis, was categorized as Hispanic, non‐Hispanic (NH) White, NH Black, NH Asian, and NH other. NH other includes those who are American Indian or Alaskan Native (AIAN), Native Hawaiian or Other Pacific Islander (NHOPI), and those who chose more than one race. This single category was created given the relatively small numbers of the subgroups within that category.

The primary outcome for this analysis is PTB, defined as GA at delivery <37 weeks. Secondary outcomes included subcategories of PTB, including (1) extremely preterm (<29 weeks), (2) moderately preterm (29 to 33 weeks and 6 days), and (3) late preterm (34 to 36 weeks and 6 days) birth.

A guide on the definition of each variable for all the included years can be found in the Vital Statistics Online Data Portal (https://www.cdc.gov/nchs/data_access/vitalstatsonline.htm).

### 2.4 Statistical analysis

Descriptive statistics were used to summarize and compare newborn and maternal characteristics for each nativity, ethnicity, and racial subgroup using percent for categorical variables and mean with standard deviation (SD) for continuous variables. Maternal characteristics for each GA category are displayed using percentages and means (SD). The incidence of PTB overall and by GA category was described for each nativity, ethnicity, and race subgroup. We performed binary (for preterm [<37 weeks vs. ≥37 weeks]) and multinomial logistic regression (for preterm categories [< 29, 29–< 34, 34–< 37 weeks using ≥37 weeks as reference]) analyses to calculate adjusted odds ratios (aORs) and 95% confidence intervals (CIs) to quantify the associations between maternal nativity status and PTB for all self‐reported maternal race and ethnic group and stratified by each self‐reported maternal race and ethnic group. We used US‐born individuals as the reference group in the analyses, as this was the largest population, which allowed for more stable estimates in the regression models. We selected within‐group comparisons (e.g., non–US born vs US born within each racial/ethnic group) to reduce confounding by structural and sociodemographic differences across groups. While alternative approaches using a single reference group are possible, this approach allows for more interpretable assessment of nativity effects within socially defined populations. Adjustment covariates included fixed effects for delivery year and covariates known for their clinical association with PTB, which were maternal age, education (five‐level), insurance (three‐level), prenatal care, smoking in the first trimester, prepregnancy DM, prepregnancy HTN, prepregnancy BMI, and year of birth (eight‐level). We performed binary logistic regression using all race and ethnicity groups combined. We then tested for interaction effects between nativity and race/ethnicity to evaluate whether the association between maternal nativity and PTB varied across racial and ethnic groups, and repeated the binary logistic regression stratifying by race and ethnicity groups if the interaction term was significant. Multinomial regression models were performed to assess the relationship between nativity and the PTB categories. Forest plots were used to visually summarize the aORs and 95% CIs for binomial and multinomial models. Ordinal logistic regression was considered; however, the proportional odds assumption was not met; therefore, multinomial logistic regression was used. For all analyses, two‐sided *p*  <  0.05 indicated statistical significance. Statistical analysis was performed using SAS (Version 9.4; SAS Institute).

## 3 RESULTS

A total of 29,950,846 births were initially identified from 2016 to 2023. After exclusions, the final analytic sample included 467,446 twin births (Figure 1). Maternal and obstetrical characteristics stratified by GA at delivery are presented in Table 1. In the study sample, 184,692 births (39.5%) occurred at term (≥37 weeks), while 282,754 (60.5%) were preterm (<37 weeks). Among the PTB, 191,972 (41.1%) were late preterm (34–< 37 weeks), 68,457 (14.6%) were moderately preterm (29–<34 weeks), and 22,325 (4.8%) were extremely preterm (<29 weeks) (Table 1). The database does not include data on whether preterm deliveries were indicated or spontaneous. Maternal and obstetrical characteristics stratified by ethnicity and nativity are provided in Table S1.

**FIGURE 1:**
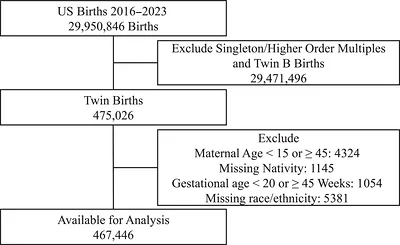
Flow diagram of the study population.

**TABLE 1:** Demographics and medical characteristics by gestational age at delivery 2016–2023.

	Term ≥37 weeks	Late preterm 34–<37 weeks	Moderate preterm 29–<34 weeks	Extreme preterm <29 weeks
	*N* = 184,692	*N* = 191,972	*N* = 68,457	*N* = 22,325
Maternal nativity				
US born	146,771 (79.5%)	156,698 (81.6%)	56,691 (82.8%)	18,504 (92.9%)
Non–US born	37,921 (20.5%)	35,274 (18.4%)	11,766 (17.2%)	3821 (17.1%)
Maternal age				
≤19	2962 (1.6%)	4499 (2.3%)	2294 (3.4%)	1023 (4.6%)
20–35	135,033 (73.1%)	142,138 (74.0%)	51,025 (74.5%)	16,926 (75.8%)
≥35	46,697 (25.3%)	45,335 (23.6%)	15,138 (22.1%)	4376 (19.6%)
Maternal education				
<High school	15,343 (8.4%)	17,455 (9.2%)	7256 (10.7%)	2557 (11.6%)
High school diploma	39,697 (21.7%)	45,172 (23.7%)	17,628 (26.0%)	6375 (29.0%)
Any college	50,037 (27.3%)	54,665 (28.7%)	19,714 (29.1%)	6627 (30.2%)
Bachelor's degree	46,526 (25.4%)	43,980 (23.1%)	14,208 (21.0%)	3970 (18.1%)
Advanced degree	31,428 (17.2%)	29,037 (15.3%)	8973 (13.2%)	2439 (11.1%)
Parity				
Parous	135,419 (73.3%)	135,814 (70.8%)	45,587 (66.6%)	14,291 (64.0%)
Nulliparous	49,273 (26.7%)	56,158 (29.3%)	22,870 (33.4%)	8034 (36.0%)
Prepregnancy hypertension				
Yes	4646 (2.5%)	7616 (4.0%)	3505 (5.1%)	1209 (5.4%)
No	179,905 (97.5%)	184,214 (96.0%)	64,868 (94.9%)	21,078 (94.6%)
Prepregnancy diabetes				
Yes	1347 (0.7%)	2346 (1.2%)	1275 (1.9%)	414 (1.9%)
No	183,204 (99.3%)	189,484 (98.8%)	67,098 (98.1%)	21,873 (98.1%)
Maternal BMI				
Mean (SD)	29.7 (12.3)	29.9 (12.6)	30.6 (14.4)	32.2 (16.8)
Smoking (first trimester)				
Yes	8154 (4.4%)	10,564 (5.5%)	4790 (7.0%)	1616 (7.3%)
No	175,875 (95.6%)	180,705 (94.5%)	63,303 (93.0%)	20,553 (92.7%)
Insurance				
Medicaid	65,073 (35.8%)	74,580 (39.1%)	29,154 (42.8%)	10,143 (45.8%)
Private	105,099 (57.2%)	105,011 (55.0%)	34,957 (51.4%)	10,533 (47.5%)
Other	12,875 (7.0%)	11,297 (5.9%)	3955 (5.8%)	1494 (6.7%)
Prenatal care				
Yes	1895 (1.0%)	2619 (1.4%)	2026 (3.1%)	1285 (6.2%)
No	179,458 (99.0%)	184,420 (98.6%)	63,442 (96.9%)	19,554 (93.8%)

Abbreviation: BMI, body mass index.

*N* = 467,446 twins.

To evaluate whether the association between maternal nativity and PTB varied across racial and ethnic groups, we tested for interaction effects between nativity and race/ethnicity in both binary and multinomial logistic regression models. The type III p‐value for the interaction term was 0.03 in the binary model, indicating a statistically significant interaction. In the multinomial model, which categorized PTB into three GA subgroups, the type III *p* value was <0.0001, suggesting strong evidence that the effect of nativity on PTB differs significantly by race and ethnicity. Consequently, further stratified analyses were conducted.

Table 2 describes PTB rates overall of less than 37 weeks and by GA subgroup (<29, 29–<34, and 34–<37 weeks) across race/ethnic groups, stratified by nativity. Among US‐born individuals, the overall PTB rate (<37 weeks) was 61.8% for Hispanic, 65.6% for Black, 61.6% for Asian, 61.7% for Other, and 59.8% for White individuals. Among non‐US‐born individuals, PTB rates were generally lower: 60.2% for Hispanic, 54.0% for Black, 57.0% for Asian, 58.0% for Other, and 53.6% for White individuals.

**TABLE 2:** Distribution of preterm birth overall and by gestational age categories (<29, 29–<34, and 34–<37 weeks) stratified by maternal nativity and race/ethnicity (*n* = 467,446).

Maternal nativity, ethnicity, and race	Term ≥37 weeks	Preterm <37 weeks	Extremely <29 weeks	Moderately 29–<34 weeks	Late 34–<37 weeks
All	184,692 (39.5 %)	282,754 (60.5 %)	22,325 (4.8 %)	68,457 (14.6 %)	191,972 (41.1 %)
Non‐US‐born Hispanic	15,175 (39.8 %)	22,955 (60.2 %)	1768 (4.6 %)	5475 (14.4 %)	15,712 (41.2 %)
US‐born Hispanic	18,943 (38.2 %)	30,665 (61.8 %)	2566 (5.2 %)	7497 (15.1 %)	20,602 (41.5 %)
Non‐US‐born Black	6017 (46.0 %)	7076 (54.0 %)	788 (6.0 %)	1639 (12.5 %)	4649 (35.5 %)
US‐born Black	25,008 (34.4 %)	47,650 (65.6 %)	5843 (8.0 %)	12,462 (18.2 %)	29,345 (44.3 %)
Non‐US‐born Asian	8171 (43.0 %)	10,811 (57.0 %)	706 (3.7 %)	2398 (12.6 %)	7707 (40.6 %)
US‐born Asian	1914 (38.4 %)	3071 (61.6 %)	229 (4.6 %)	693 (13.9 %)	2149 (43.1 %)
Non‐US‐born Other	659 (42.0 %)	910 (58.0 %)	53 (3.4 %)	225 (14.3 %)	632 (40.3 %)
US‐born Other	5198 (38.3 %)	8368 (61.7 %)	677 (5.0 %)	2098 (11.9 %)	5593 (41.2 %)
Non‐US‐born White	7899 (46.4 %)	9109 (53.6 %)	506 (3.0 %)	2029 (11.9 %)	6574 (38.7 %)
US‐born White	95,708 (40.2 %)	142,139 (59.8 %)	9189 (3.9 %)	33,941 (14.3 %)	99,009 (41.6 %)

Adjusted ORs (95% CI) for PTB by maternal nativity, ethnicity, and race, adjusted for maternal age, education, insurance, prenatal care, smoking in the first trimester, prepregnancy diabetes, gestational diabetes, prepregnancy HTN, gestational HTN, prepregnancy BMI, induction of labor, and year of birth, are shown in Table 3. Non‐US‐born individuals had significantly lower overall odds of PTB compared to US‐born (OR, 0.88; 95% CI, 0.86–0.89). Stratification by PTB categories also revealed non‐US‐born individuals had lower odds of PTB compared to US‐born individuals for extremely PTB (OR, 0.85; 95% CI, 0.81–0.88) (Figure 2), moderately PTB (OR, 0.82; 95% CI, 0.80–0.84) (Figure 3), and late PTB (OR, 0.90; 95% CI: 0.88–0.92) (Figure 4).

**TABLE 3:** Adjusted odds ratio (95% confidence interval) for preterm delivery by race/ethnicity and maternal nativity.

Maternal nativity, ethnicity, and race	Preterm <37 weeks	Extremely <29 weeks	Moderately 29–<34 weeks	Late 34–<37 weeks
All combined models				
Non–US born (All)	0.89 (0.88–0.91)	0.88 (0.84–0.91)	0.85 (0.83–0.87)	0.91 (0.89–0.93)
US born (All)	1 (ref)	1 (ref)	1 (ref)	1 (ref)
Stratified by race/ethnicity				
Non‐US‐born Hispanic	0.93 (0.90–0.96)	0.86 (0.80–0.93)	0.90 (0.85–0.94)	0.95 (0.91–0.98)
US‐born Hispanic	1 (ref)	1 (ref)	1 (ref)	1 (ref)
Non‐US‐born Black	0.65 (0.62–0.68)	0.61 (0.56–0.67)	0.58 (0.54–0.62)	0.69 (0.65–0.72)
US‐born Black	1 (ref)	1 (ref)	1 (ref)	1 (ref)
Non‐US‐born Asian	0.87 (0.81–0.93)	0.81 (0.68–0.96)	0.87 (0.78–0.96)	0.87 (0.81–0.94)
US‐born Asian	1 (ref)	1 (ref)	1 (ref)	1 (ref)
Non‐US‐born Other	0.90 (0.80–1.01)	0.75 (0.55–1.03)	0.90 (0.76–1.07)	0.92 (0.81–1.04)
US‐born Other	1 (ref)	1 (ref)	1 (ref)	1 (ref)
Non‐US‐born White	0.85 (0.82–0.88)	0.78 (0.71–0.87)	0.81 (0.77–0.86)	0.87 (0.84–0.90)
US‐born White	1 (ref)	1 (ref)	1 (ref)	1 (ref)

Abbreviation: BMI, body mass index.

Adjusted for maternal age, education, insurance, prenatal care, smoking first trimester, prepregnancy diabetes, prepregnancy hypertension, prepregnancy BMI, and year of birth.

**FIGURE 2:**
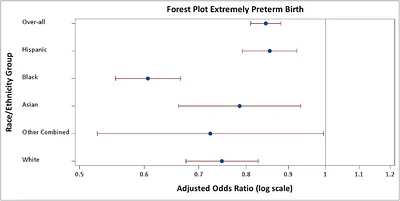
Forest plot displaying the adjusted odds ratios (aORs) and 95% confidence intervals (CIs) for extremely preterm birth (<29 weeks’ gestation) across nativity, race/ethnicity groups.

**FIGURE 3:**
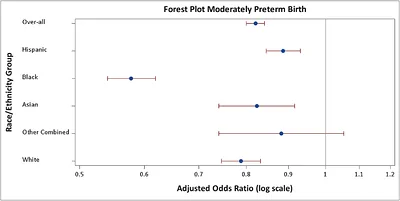
Forest plot displaying the adjusted odds ratios (aORs) and 95% confidence intervals (CIs) for moderately preterm birth (29–<34 weeks’ gestation) across nativity, race/ethnicity groups.

**FIGURE 4:**
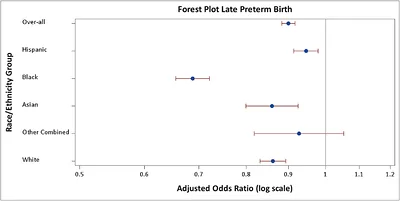
Forest plot displaying the adjusted odds ratios (aORs) and 95% confidence intervals (CIs) for late preterm birth (34–<37 weeks’ gestation) across nativity, race/ethnicity groups.

Statistically significant differences were observed among specific ethnic and racial groups across all categories of PTB. Stratified by race and ethnicity, non‐US‐born Black individuals exhibited the lowest odds of PTB (OR, 0.65; 95% CI, 0.62–0.68) relative to their US‐born Black counterparts. Non‐US‐born Black individuals also exhibited the lowest odds in comparison to their US‐born counterparts across all GA categories: extremely preterm (OR, 0.61; 95% CI, 0.55–0.67), moderately preterm (OR, 0.58; 95% CI, 0.54–0.62), and late preterm (OR, 0.69; 95% CI, 0.66–0.72).

## 4 DISCUSSION

Our study demonstrates that maternal nativity is associated with PTB in twin pregnancies, with non‐US‐born individuals showing an 12% lower odds of PTB compared to their US‐born counterparts. This protective association was consistent across all PTB subcategories and was most pronounced among Black individuals. These findings align with prior research of singleton pregnancies, which similarly found lower PTB rates among non‐US‐born women [11, 12]. A nationwide study of 8.6 million first‐time births in the US reported significantly lower PTB rates among non‐US‐born individuals across racial and ethnic groups, with the greatest risk reduction among non‐Hispanic Black individuals [11]. Similarly, a study of 4.5 million births in Pennsylvania confirmed a protective effect of non‐US birth status on PTB risk across racial groups [20]. Even after adjusting for a variety of socioeconomic and health covariates, US‐born Black individuals remained at higher risk of adverse birth outcomes compared to non‐US‐born Black pregnancies [21]. Our study extends this evidence to twin pregnancies, a group at inherently higher risk for PTB.

It is well established that there are PTB disparities by race and ethnicity in the United States [22, 23, 24]. Interestingly, the comparable PTB rates observed in this study between non‐US‐born Black and White women suggest that social and structural factors linked to nativity, rather than race alone, contribute meaningfully to odds differences. Prior studies have also shown higher rates of prematurity and neonatal mortality among twins born to US‐born Black mothers compared with those born to non‐US‐born Black mothers [25]. Although our data do not directly assess mechanistic pathways, the association between nativity and PTB likely reflects multiple overlapping influences beyond structural inequities and interpersonal racism, including cultural, behavioral, and migration‐related factors.

Nativity is an important social determinant of health that may serve as a proxy for broader socioenvironmental and behavioral factors [11]. The underlying factors contributing to these nativity‐based disparities are complex and multifactorial. The “healthy immigrant” effect suggests that individuals who migrate may represent a self‐selected healthier subgroup, possibly due to selective migration patterns favoring those with better baseline health, stronger social networks, and resilience, leading to more favorable pregnancy outcomes [25]. Additionally, non‐US‐born mothers may initially maintain cultural norms and values that promote healthier lifestyles and strong familial and social support [26, 27, 28]. However, these protective factors may erode with prolonged US residence due to acculturation, increased stress exposure, and lifestyle changes [29]. The deterioration of perinatal health outcomes in subsequent generations is thought to reflect the cumulative impact of systemic barriers, chronic stress, and limited access to resources that support healthy living, all of which may accelerate physiologic aging [30, 31, 32].

The substantially lower odds of PTB among non‐US‐born Black pregnancies compared to US‐born counterparts are consistent with prior literature, but the mechanisms underlying this difference remain unclear. Our data do not directly assess these pathways, but they raise important questions about whether being US born may serve as a risk marker or proxy for the lived experience of Black Americans, including the effects of structural racism and discrimination. Future research should explore the underlying drivers of PTB disparities among US‐born Black individuals to guide targeted and effective interventions.

Maternal stress is a well‐documented risk factor for PTB [33]. It can lead to neuroendocrine and immune‐inflammatory changes, including increased levels of corticotropin‐releasing hormone (CRH), cortisol, and proinflammatory cytokines, which can contribute to premature activation of labor pathways [34]. National data indicate that non‐US‐born individuals experience lower rates of psychiatric disorders, including anxiety, depression, and trauma‐related conditions, compared with their US‐born counterparts [35].

Potential mechanisms underlying these findings may include cumulative physiologic stress, often conceptualized as allostatic load; however, our dataset does not include direct measures of stress, discrimination, or duration of residence in the United States. Therefore, these explanations should be interpreted as hypotheses.

These findings likely reflect multiple mechanisms, including the healthy immigrant effect, cultural and behavioral factors, differences in healthcare utilization, and potential selection effects among migrants. While structural racism and discrimination may contribute, these should be interpreted as hypotheses.

Given that nativity is associated with PTB, it warrants consideration in clinical risk assessments and public health initiatives. Tailored prenatal care models incorporating culturally sensitive approaches may help sustain protective maternal health behaviors while mitigating the adverse effects of acculturation. Additionally, addressing structural barriers to equitable healthcare access, particularly for Black individuals, remains crucial for reducing disparities in PTB outcomes.

Lastly, nativity may serve as a population‐level risk marker rather than an individual‐level clinical predictor, and its incorporation into clinical care should be interpreted cautiously. These findings demonstrate an association between maternal nativity and PTB and do not establish causality.

This study has several strengths, including the use of a large, nationally representative dataset with detailed demographic and clinical information, allowing for robust statistical adjustments and subgroup analyses. Furthermore, the stratified analysis by race and ethnicity allows for a nuanced understanding of how nativity influences PTB odds across different racial and ethnic subgroups. Notably, the identification of the strongest protective effect in non‐US‐born Black mothers adds to existing evidence supporting the healthy immigrant effect, but also highlights the influence of social and structural determinants of health that drive disparities for US‐born Black pregnancies [36].

Despite these strengths, several limitations should be acknowledged. A key limitation is that our data do not differentiate between spontaneous and medically indicated PTBs, which is particularly important in twin pregnancies, where a substantial proportion of preterm deliveries are clinically indicated and may differ systematically by maternal characteristics. This limitation is particularly relevant in the context of twin gestations, where early delivery is often planned for clinical reasons, such as monochorionic‐diamniotic twins, given the lack of data on chorionicity in the dataset. The dataset fails to capture important acculturation factors, such as the length of residence in the United States, generational status, which may be highly relevant as the protective effect associated with non‐US nativity may attenuate with longer duration of exposure to US social and environmental conditions, and paternal nativity, which might influence the observed differences in PTB odds. Without these details, it remains unclear whether the protective effects seen among non‐US‐born individuals persist over time or decline with greater exposure to US‐specific environmental and social factors. Furthermore, although the study adjusts for significant medical and sociodemographic factors, unmeasured confounders like maternal stress, discrimination, diet, and environmental exposures may still contribute to nativity‐related disparities in PTB. The “healthy immigrant” effect, wherein individuals who migrate may constitute a self‐selected, healthier subgroup, might also help explain the lower PTB risk seen among non‐US‐born mothers. Relying on CDC natality data introduces additional limitations, as self‐reported race and ethnicity could be prone to misclassification bias. While the study accounts for numerous potential confounders, residual confounding may still affect the results.

## CONFLICT OF INTEREST STATEMENT

The authors report no conflicts of interest.

## FUNDING INFORMATION

The authors received no specific funding for this work.

## Supporting information



Supporting Information

## Data Availability

The data that support the findings of this study are openly available in Vital Statistics Online Data Portal at https://www.cdc.gov/nchs/data_access/vitalstatsonline.htm.
