# Supplementary material for: Maternal nativity and preterm birth disparities in twin pregnancies: A nationwide population‐based study
Source: Pregnancy (Hoboken). 2026 Jul 20;2(4):e70361. doi: 10.1002/pmf2.70361 (PMC13624835; doi:10.1002/pmf2.70361)
Supplement: Supplementary file 1 [file PMF2-2-e70361-s001.docx]

**Table 1.** Demographics and Medical Characteristics by Maternal Nativity, Ethnicity and Race from 2016-2023. N=467,446 Twins

|  |  | | Non-Hispanic | | | | | | | |
| --- | --- | --- | --- | --- | --- | --- | --- | --- | --- | --- |
|  | Hispanic  87,738 (18.8%) | | Black  85,751 (18.3%) | | Asian  23,967 (5.1%) | | Other  15,135 (3.2%) | | White  254,855 (54.5%) | |
| Maternal Nativity | Non-US  Born  38,130 (43.5%) | US-  Born  49,608 (56.5%) | Non-US  Born  13,093 (15.3%) | US-  Born  72,658 (84.7%) | Non-US  Born  18,982 (79.2%) | US-  Born  4,985 (20.8%) | Non-US  Born  1,569 (10.4%) | US-  Born  13,566 (89.6%) | Non-US  Born  17,008 (6.7%) | US-  Born  237,847 (93.3%) |
| Maternal Age  ≤19  20-35  ≥35 | 920 (2.4%)  25,702 (67.4%)  11,508 (30.2%) | 2,408 (4.9%)  39,041 (78.7%)  8,159 (16.5%) | 63 (0.5%)  8,295 (63.4%)  4,735 (36.2%) | 3,051 (4.2%)  58,714 (80.8%)  10,893 (15.0%) | 33 (0.2%)  11,646 (61.4%)  7.303 (38.5%) | 28 (0.6%)  2,955 (59.3%)  2,002 (40.2%) | 31 (2.0%)  1,094 (69.7%)  444 (28.3%) | 591 (4.4%)  10,690 (78.8%)  2,285 (16.8%) | 76 (0.5%)  10,425 (61.3%)  6,507 (38.3%) | 3,577 (1.5%)  176,560 (74.2%)  57,710 (24.3%) |
| Maternal Education  <High school  High school diploma  Any college  Bachelor’s degree  Advanced degree | 11,524 (30.9%)  11,308 (30.3%)  7,287 (19.5%)  4,860 (13.0%)  2,318 (6.2%) | 5,873 (12.0%)  15,567 (31.7%)  17,029 (34.7%)  7,117 (14.5%)  3,473 (7.1%) | 1,589 (12.3%)  3,037 (23.5%)  3,326 (25.8%)  3,115 (24.1%)  1,846 (14.3%) | 7,817 (10.9%)  26,821 (37.2%)  25,128 (34.9%)  7,680 (10.7%)  4,614 (6.4%) | 1,063 (5.7%)  1,928 (10.4%)  2,533 (13.6%)  6,651 (35.7%)  6,435 (34.6%) | 82 (1.7%)  367 (7.5%)  871 (17.8%)  1,778 (36.2%)  1,808 (36.9%) | 218 (14.2%)  351 (22.8%)  440 (28.6%)  321 (20.9%)  207 (13.5%) | 1,519 (11.3%)  3,922 (29.2%)  4,862 (36.1%)  2,027 (15.1%)  1,126 (8.4%) | 996 (6.0%)  2,390 (14.3%)  3,258 (19.5%)  5,590 (33.5%)  4,463 (33.5%) | 11,930 (5.0%)  43,181 (18.3%)  66,309 (28.0%)  69,545 (29.4%)  45,587 (19.3%) |
| Parous  Nulliparous | 28,900 (75.8%)  9,230 (24.2%) | 35,374 (71.3%)  14,234 (28.7%) | 9,821 (75.0%)  3,272 (25.0%) | 57,051 (78.5%)  15,607 (21.5%) | 10,579 (55.7%)  8.403 (44.3%) | 2,923 (58.6%)  2,062 (41.4%) | 1,138 (72.5%)  431 (27.5%) | 10,029 (73.9%)  3,537 (26.1%) | 10,977 (64.5%)  6,031 (35.5%) | 164,319 (69.1%)  73,528 (30.9%) |
| Pre-Pregnancy Hypertension, Yes  No | 840 (2.2%)  37,258 (97.8%) | 1,271 (2.6%)  48,311 (97.4%) | 495 (3.8%)  12,584 (96.2%) | 5,038 (6.9%)  67,555 (93.1%) | 305 (1.6%)  18,665 (98.4%) | 125 (2.5%)  4,855 (97.5%) | 33 (2.1%)  1,535 (97.9%) | 571 (4.2%)  12,982 (95.8%) | 251 (1.5%)  16,738 (98.5%) | 8,047 (3.4%)  229,582 (96.6%) |
| Pre-Pregnancy Diabetes, Yes  No | 513 (1.4%)  37,585 (98.7%) | 610 (1.2%)  48,972 (98.8%) | 183 (1.4%)  12,896 (98.6%) | 1,119 (1.5%)  71,474 (98.5%) | 254 (1.3%)  18,716 (98.7%) | 68 (1.4%)  4,912 (98.6%) | 16 (1.0%)  1,552 (99.0%) | 199 (1.5%)  13,354 (98.5%) | 110 (0.7%)  16,879 (99.4%) | 2,310 (1.0%)  235,319 (99.0%) |
| Mean(SD) Maternal BMI | 27.7 (5.87) | 29.7 (7.30) | 28.0 (6.01) | 31.1 (8.32) | 24.1 (4.58) | 25.6 (5.43) | 27.8 (6.81) | 29.8 (7.91) | 25.3 (5.42) | 27.9 (7.18) |
| Cigarettes 1^st^ Trimester, Yes  No | 149 (0.4%)  37,861 (99.6%) | 1,264 (2.6%)  48,179 (97.4%) | 44 (0.3%)  13,006 (99.7%) | 4,049 (5.6%)  68,140 (94.4%) | 24 (0.1%)  18,916 (99.9%) | 39 (0.8%)  4,935 (99.2%) | 32 (2.1%)  1,526 (98.0%) | 1,417 (10.5%)  12,035 (89.5%) | 222 (1.3%)  16,734 (98.7%) | 17,884 (7.6%)  219,104 (92.5%) |
| Insurance  Medicaid  Private  Other | 21,637 (57.0%)  10,483 (27.6%)  5,812 (15.3%) | 26,096 (52.9%)  20,760 (42.1%)  2,517 (5.1%) | 6,523 (50.2%)  4,724 (36.4%)  1,736 (13.4%) | 48,379 (66.9%)  20,902 (28.9%)  2,995 (4.1%) | 4,032 (21.3%)  13,109 (69.4%)  1,755 (9.3%) | 682 (13.7%)  4,092 (82.4%)  191 (3.9%) | 682 (44.0%)  685 (44.2%)  182 (11.8%) | 6,975 (51.8%)  5,510 (40.9%)  986 (7.3%) | 5,113 (30.2%)  10,254 (60.7%)  1,541 (9.1%) | 59,461 (25.2%)  165,081 (69.8%)  11,906 (5.0%) |
| No Prenatal Care  Prenatal Care | 778 (2.1%)  36,258 (97.9%) | 853 (1.8%)  47,473 (98.2%) | 301 (2.4%)  12,270 (97.6%) | 2,192 (3.1%)  67,869 (96.9%) | 157 (0.9%)  18,341 (99.2%) | 52 (1.1%)  4,813 (98.9%) | 52 (3.4%)  1,467 (96.6%) | 353 (2.7%)  12,814 (97.3%) | 225 (1.4%)  16,278 (98.6%) | 2,862 (1.2%)  229,291 (98.8%) |
| Gestational Age at Delivery  < 24w  24 < 34w  34 < 37w  37w+ | 1,768 (4.6%)  5,475 (4.6%)  15,712 (41.2%)  15,175 (39.8%) | 2,566 (5.2%)  7,497 (15.1%)  20,602 (41.5%)  18,943 (38.2%) | 788 (6.0%)  1,639 (12.5%)  4,649 (35.5%)  6,017 (46.0%) | 5,843 (8.0%)  12,462 (17.2%)  29,345 (40.4%)  25,008 (34.4%) | 706 (3.7%)  2,398 (12.6%)  7,707 (40.6%)  8,171 (43.1%) | 229 (4.6%)  693 (13.9%)  2,149 (43.1%)  1,914 (38.4%) | 53 (3.4%)  225 (14.3%)  632 (40.3%)  659 (42.0%) | 677 (5.0%)  2,098 (15.5%)  5,593 (41.2%)  5,198 (38.3%) | 506 (3.0%)  2,029 (11.9%)  6,574 (11.9%)  7,899 (46.4%) | 9,189 (3.9%)  33,941 (14.3%)  99,009 (41.6%)  95,708 (40.2%) |
